# The Kinetics of Formation of Microporous Polytriazine in Diphenyl Sulfone

**DOI:** 10.3390/molecules27113605

**Published:** 2022-06-03

**Authors:** Andrey Galukhin, Ilya Nikolaev, Roman Nosov, Sergey Vyazovkin

**Affiliations:** 1 Physical Chemistry Department, Alexander Butlerov Institute of Chemistry, Kazan Federal University, Kremlevskaya Str. 18, 420008 Kazan, Russia; ilkamoe1995@yandex.ru (I.N.); romanosow@mail.ru (R.N.); 2 Department of Chemistry, University of Alabama at Birmingham, 901 S. 14th Street, Birmingham, AL 35294, USA

**Keywords:** cyanate esters, polymerization kinetics, microporous polymers, polytriazines, differential scanning calorimetry, isoconversional kinetic analysis

## Abstract

This study highlights the value of nonisothermal kinetic methods in selecting temperature conditions for the isothermal preparation of microporous polymeric materials. A dicyanate ester is synthesized and the kinetics of its polymerization in diphenyl sulfone are studied by calorimetry under nonisothermal conditions. The kinetics are analyzed by a model-based approach, using the Kamal model, as well as by a model-free approach, using an advanced isoconversional method. Both approaches correctly predict the time to completion of polymerization at a given temperature. The material prepared independently at the predicted temperature is characterized by electron microscopy and CO_2_ adsorption measurements and is confirmed to possess a microporous structure with a multimodal distribution of micropores with two major maxima at ~0.5 and 0.8 nm.

## 1. Introduction

Microporous (i.e., with pore sizes smaller than 2 nm [1]) organic polymers (MOPs) are promising materials for gas separation and storage, decontamination, controlled drug release, catalysis, etc. [2,3]. They combine chemical and thermal stability, high specific surface area, and the possibility of pore surface modification. Based on polymer structure, MOPs can be divided into crystalline and amorphous categories. Crystalline MOPs can be synthesized by reversible reactions, such as the dehydration of boronic acids, the trimerization of nitriles, and the condensation of amines with aldehydes [4]. The ordered porous structures of crystalline MOPs result in a narrow pore-size distribution that makes them particularly useful for molecular sieving processes. Amorphous MOPs are more universal from the processing point of view. They can be obtained by means of any polymerization reaction and are usually characterized by a wider pore-size distribution [5]. Large surface energy and high capillary pressure in amorphous MOPs stimulate the mobility of the polymer chains located near the surface that may lead to the collapse of micropores. Thus, to prevent this and make the micropores more stable, it is advised to employ polymers with high cross-linking densities, rigid polymer chains, and bulky fragments in the main chain or as side groups [6].

Polytriazines can be employed for the production of both crystalline and amorphous MOPs. Reversible cyclotrimerization of some aromatic nitriles in ionothermal conditions (e.g., in the melt of ZnCl_2_) results in the formation of highly crystalline microporous polymers with well-developed surfaces [7,8,9]. Irreversible solvothermal polymerization of cyanate esters produces amorphous polytriazines [10,11,12,13,14]. The structure of the initial monomers and conditions of their polymerization strongly affect the adsorption properties of the final material. Yet, probably the most important condition for obtaining stable micropores is attaining a practically complete extent of crosslinking polymerization. This condition is determined by the process kinetics. Although, microporous polytriazines are well known, to the best of our knowledge, the kinetics of their formation has never been studied. However, such studies are important from both fundamental and applied perspectives. Fundamentally, they help to understand how the reaction medium (solvent) affects the polymerization process (the monomer’s chemical reactivity, diffusional characteristics, and the mechanism of the reaction in general). From the applied perspective, they provide information that allows one to control polymerization and, specifically, to secure its completion.

In this paper, we synthesize a rigid dicyanate ester monomer and study the kinetics of its solvothermal polymerization in diphenyl sulfone in detail. In particular, we employ two different kinetic analyses (model-based and model-free) and test their capabilities for predicting the completion of the polymerization process. We also provide some basic characterization of the obtained porous material. The objective of this work is to highlight the usefulness of kinetic studies in optimizing the temperature conditions of the synthesis of microporous polytriazines. 

## 2. Materials and Methods

4,4′-dihydroxybiphenyl (97%, Sigma-Aldrich, Saint Louis, MI, USA), triethylamine (>99%, Fisher Scientific, Waltham, MA, USA), cyanogen bromide (97%, Acros Organics, Waltham, MA, USA), Na_2_SO_4_ (anhydrous, >99.5%, Chimmed, Moscow, Russia), dichloromethane (>99%, EKOS-1, Moscow, Russia), tetrahydrofuran (>99%, EKOS-1, Moscow, Russia), hexane (>99.5 %, EKOS-1, Moscow, Russia), diphenyl sulfone (97%, Acros Organics, Waltham, MA, USA), P_2_O_5_ (>98%, Vekton, Saint-Petersburg, Russia), SiO_2_ (60 Å, Machery-Nagel, Duren, Germany) were purchased and used without additional purification. The acetone (>98%, Chimmed, Moscow, Russia) was distilled over P_2_O_5_ before using. Arium mini instrument (Sartorius, Goettingen, Germany) was used for the preparation of deionized water (18.2 MΩ).

**Figure 1:**
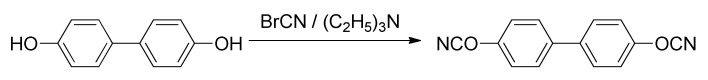
Scheme of synthesis of target dicyanate ester.

*Synthesis of target cyanate ester.* A mixture of cyanogen bromide (12.51 g, 0.118 mol) and 4,4′-dihydroxybiphenyl (10 g, 0.054 mol) in anhydrous acetone was stirred at −30 °C; a solution of triethylamine (16.42 mL, 0.118 mol) in acetone was added dropwise to the aforementioned cooled mixture (Figure 1). Resulting mixture was stirred for 20 min, then precipitated salt of triethylammonium bromide was filtered off and acetone was evaporated. Dichloromethane was added to a reaction mixture, followed by washing with deionized water and drying over anhydrous sodium sulfate. The crude product was then purified by column chromatography using dichloromethane as an eluent and subsequently recrystallized from dichloromethane/hexane mixture; yield = 75%. Melting point (DSC, averaged over heating rates): 137.6 °C. IR (cm^−1^): 2269, 2237 (-OCN functional group). ^1^H NMR (CDCl_3_–d1) δ(ppm): 7.63–7.62 (d, ArH, 4H, *J* = 8.73 Hz), 7.41–7.40 (d, ArH, 4H, *J* = 8.73 Hz). ^13^C NMR (CDCl_3_–d1) δ(ppm): 152.80, 138.47, 129.30, 116.09, 108.67.

*Methods for the determination of the structure of target monomer.* The ^1^H and ^13^C NMR experiments were carried on a Bruker AVANCE III NMR spectrometer operating at 600.13 MHz. IR spectra were recorded with Bruker Vertex 70 FTIR spectrometer.

*Preparation of a 20 wt% solution of the target cyanate ester in diphenyl sulfone*. Briefly, 80 mg of cyanate ester and 320 mg of diphenyl sulfone were melted together at 140 °C in a closed vial with a magnetic stirrer until the formation of clear solution. After that, the resulting solution was additionally stirred for 5 min. After cooling to room temperature, the mixture was carefully crushed with a metal spatula in a vial to a powder, which was then used for DSC measurements.

*Preparation of porous polymer*. Diphenyl sulfone and target dicyanate ester were weighed in a glass ampule. The glass ampule was then flushed with argon and hermetically sealed. The obtained mixture was heated to 200 °C to form a clear solution and then was placed to the preheated to 300 °C muffle furnace and polymerized for 55 min. After polymerization, the obtained light brown sample was cooled to the room temperature, carefully crushed in an agate mortar, and washed by tetrahydrofuran in Soxhlet extractor for 48 h. Obtained porous polymer was then dried in a vacuum at 150 °C. The yield of polymer was 93%.

*Thermal analysis.* A heat flux DSC 3+ (Mettler-Toledo) was employed to run calorimetric measurements. Temperature, heat flow, and tau-lag calibrations were conducted with the aid of In and Zn standards. The experiments were performed in the atmosphere of argon flow (80 mL min^−1^) at linear heating rates (2, 4, 6, and 8 °C min ^−1^) in 40 μL aluminum pans sealed in argon atmosphere. To remove the water impurities, before sealing the samples placed in aluminum pans were kept in a vacuum desiccator containing a beaker filled with P_2_O_5_ for 1 day. The mass of the cyanate ester/diphenyl sulfone sample was ~5 mg that corresponds to ~1 mg of dicyanate ester. Temperature-modulated DSC (TMDSC) measurements were made by heating from 25 to 350 °C at the heating rate of 1 °C min^−1^ superimposed with stochastic temperature pulses of 0.5 °C amplitude and pulse time that ranged from 15 to 30 s. The mass of the cyanate ester/diphenyl sulfone sample was ~10 mg for TMDSC measurements.

*Microscopy.* Scanning electron microscopy (SEM) measurements were carried out using field-emission high-resolution scanning electron microscope Merlin Carl Zeiss. Transmission electron microscopy (TEM) images were obtained using Hitachi HT7700 Excellence. Ultramicrotome Leica EM UC7 was used for the preparation of ultrathin cuts of the porous polymer for TEM measurements.

*Adsorption measurement.* Carbon dioxide adsorption measurement was performed with ASAP 2020 MP instrument (Micromeritics) at 0 °C. Before measurement, sample was degassed by heating at 150 °C under vacuum (8 µmHg) for 5 h. Adsorption isotherm contained about 300 points. Pore-size distribution was calculated with slit-pore geometry model by means of non-local density functional theory based software (ASAP 2020 V 4.04).

## 3. Computations

Kinetic analysis was performed in accordance with the recommendations of the ICTAC Kinetics Committee [15]. The dependence of the effective activation energy, *E_α_*, on conversion was evaluated by means of the flexible integral isoconversional method of Vyazovkin. The extents of conversion, *α*, were determined as the partial areas of the DSC peaks associated with polymerization of the cyanate ester. The Vyazovkin method eliminates a systematic error in *E_α_* that arises when *E_α_* varies significantly with *α* [16]. This error is eliminated thanks to the flexible integration that presumes the constancy of *E_α_* only within a very narrow integration range, Δ*α* = 0.01. For each Δ*α*, *E_α_* is found by minimizing the following function:

(1) $$\Psi \left(E_{\alpha }\right)=\overset{p}{\underset{i=1}{\sum }}\overset{p}{\underset{j\neq i}{\sum }}\frac{J\left[E_{\alpha },T_{i}\left(t_{\alpha }\right)\right]}{J\left[E_{\alpha },T_{j}\left(t_{\alpha }\right)\right]}\;\;\;\;$$

where

(2) $$J\left[E_{\alpha },T_{i}\left(t_{\alpha }\right)\right]\;\equiv \;\int_{t_{\alpha -\Delta \alpha }}^{t_{\alpha }}\exp\left[\frac{-E_{\alpha }}{RT_{i}\left(t\right)}\right]dt\;\;\;\;$$

and *p* is the number of the temperature programs, *T*(*t*). The trapezoid rule was used to evaluate the integral. Although there are more accurate ways of integration, testing on simulated DSC data shows that the trapezoid rule permits estimating the activation energy with the average uncertainty of 0.1% [17], which is at least an order of magnitude smaller than the typical experimental uncertainty. The minimum for Equation (1) was found by the COBYLA non-gradient method from the NLopt library. The experimental uncertainties in the *E_α_* values were determined by means of a statistical procedure explained elsewhere [18].

The dependence of the pre-exponential factor on conversion was estimated by substituting the values of *E_α_* into the equation for the compensation effect:

(3) $$\ln A_{\alpha }=a+bE_{\alpha }\;\;\;\;$$

First, the *a* and *b* values were found by fitting the pairs of *lnA_i_* and *E_i_* into Equation (3). The *lnA_i_* and *E_i_* pairs were determined by substituting different reaction models, *f_i_(α)*, into the linear form of the basic rate equation:

(4) $$\ln\left(\frac{d\alpha }{dt}\right)-\ln\left[f_{i}\left(\alpha \right)\right]=\ln A_{i}-\frac{E_{i}}{RT}\;\;\;$$

Substitution of each *f_i_*(α) model into Equation (4) yielded a corresponding pair of *lnA_i_* and *E_i_* values. Overall, five pairs of *lnA_i_* and *E_i_* were determined by using the model:

(5) $$f\left(\alpha \right)=\alpha^{m}{\left(1-\alpha \right)}^{n}\;\;$$

with five different combinations of *m* and *n* (*m* = 1, *n* = 1; *m* = 0.5, *n* = 1; *m* = 1, *n* = 0.5; *m* = 2, *n* = 1; *m* = 1, *n* = 2). This model was chosen because it imitates the autocatalytic reaction kinetics typically observed for the polymerization of cyanate esters [19]. In addition, this model is a part of the Kamal reaction model [20]

(6) $$\frac{d\alpha }{dt}=k_{1}\left(T\right){\left(1-\alpha \right)}^{n}+k_{2}\left(T\right)\alpha^{m}{\left(1-\alpha \right)}^{n}$$

that has been used broadly for parameterizing the kinetics of cyanate ester polymerization [21,22].

The nonisothermal data were used to predict the polymerization kinetics under the isothermal conditions. This was performed in model-based and model-free methods. The model-based prediction was accomplished by determining the parameters of the Kamal Equation (6) and integrating it numerically at constant temperature, *T*_0_. The model-free prediction was made by employing the isoconversional technique [23], as represented by Equation (7):

(7) $$t_{\alpha }=\underset{\alpha }{\sum }\frac{J\left[E_{\alpha },T_{i}\left(t_{\alpha }\right)\right]}{exp\left(-\frac{E_{\alpha }}{RT_{0}}\right)}$$

where *t_α_* is the time to reach the conversion α at temperature, *T*_0_.

## 4. Results and Discussion

**Figure 2:**
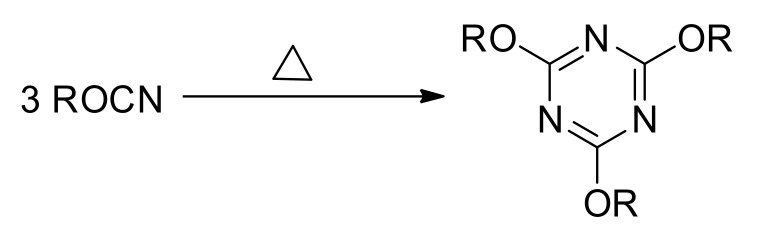
Scheme of cyclotrimerization of cyanate ester.

Thermal polymerization of cyanate esters containing more than one OCN group results in the formation of highly cross-linked polymer networks by the reaction shown in Figure 2. The formation of 1,3,5-triazine cross-links generates a significant amount of heat, which makes calorimetric techniques suitable for the monitoring of the reaction kinetics [24]. The preparation of microporous polytriazines is based on the direct synthesis methodology [6], which involves solution polymerization of an appropriate monomer in a high boiling solvent. In general, any high boiling solvent capable of dissolving cyanate esters, such as ditolylmethane, diglyme, anisole, or nitrobenzene, can be used for the solution polymerization of cyanate esters [25]. However, in most studies diphenyl sulfone is used due to its good dissolving ability toward cyanate esters and extremely high boiling point (379 °C), which allows it to perform polymerization both in non-catalytic and catalytic ways [10,11,12].

**Figure 3:**
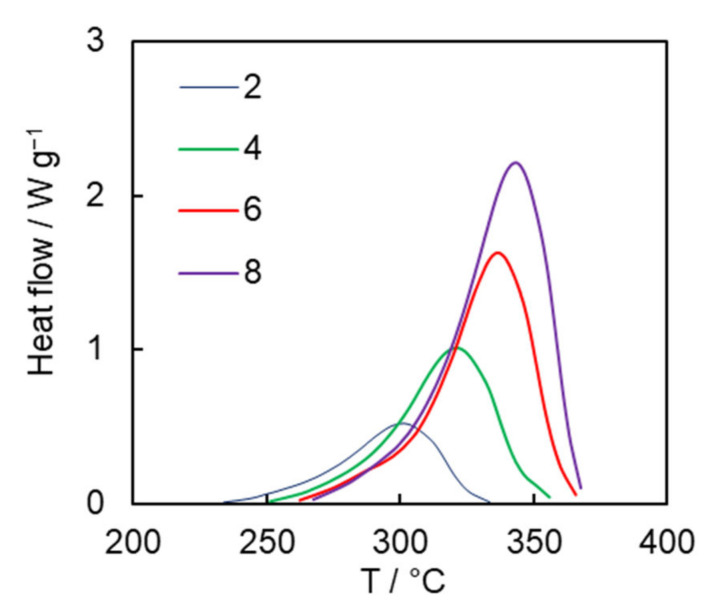
DSC curves for dicyanate ester polymerization (numbers denote heating rates in °C min^−1^).

Recently, we have shown that the reactivity of cyanate esters in diphenyl sulfone is significantly reduced compared to that in the bulk. The effect appears to arise from the preferential solvation of a cyanate ester monomer [26]. Thus, non-catalyzed solution polymerization of cyanate esters requires rather high temperatures to proceed at an acceptable rate. Figure 3 presents the DSC curves for solution polymerization of the dicyanate ester at different heating rates. The average heat of polymerization is 680 ± 10 J g^−1^, which corresponds to 80 ± 2 kJ per 1 mole of OCN groups. This value fits the range of the reaction heat values of 80–110 kJ per 1 mole of OCN groups reported for mono- and di-cyanate esters in the case of bulk polymerization [27] and closely matches the reaction heat value for solution polymerization of previously studied tricyanate esters (77 kJ per 1 mole of OCN groups) [26].

**Figure 4:**
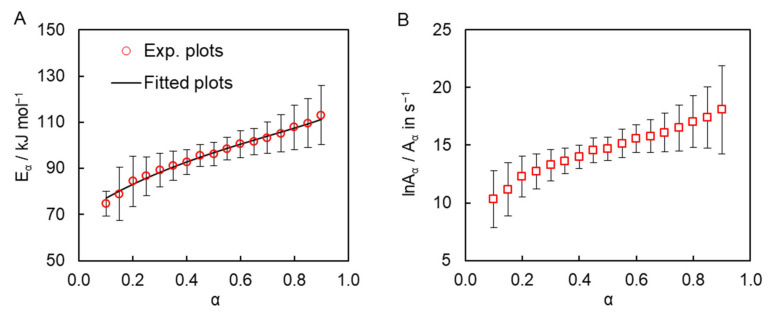
Dependence of activation energy with the best fit of Equation (9) (**A**) and pre-exponential factor (**B**) as a function of conversion for the solution polymerization of cyanate ester.

The isoconversional kinetic analysis of the measured calorimetric data was carried out to quantify the reactivity of the obtained dicyanate ester in the solution of diphenyl sulfone. The simultaneous use of the DSC data obtained at all four heating rates gives rise to the dependence of the activation energy *E_α_* and pre-exponential factor *A_α_* on conversion are presented in Figure 4. One can observe a significant systematic variation of both parameters with conversion. Such variation can be caused either by the simultaneous occurrence of several chemical reaction steps or by a transition of the process from the reaction- to diffusion-control regime. We posit that the observed *E_α_* variation is most likely caused by the multi-step nature of the process for the following three reasons. First, TMDSC measurements do not reveal any signs of vitrification of the reaction mixture during polymerization, which is broadly accepted as evidence of the transition of the polymerization process to the diffusion-controlled regime [28]. Second, the transition to the diffusion-controlled regime does not appear typical for the bulk polymerization of dicyanate esters in general [22,24,29]. Since the viscosity of the reaction medium in the solution polymerization is certainly lower than that in the bulk process, the transition to diffusion-control becomes even less probable. Third, in those cases when the transition to diffusion control does occur in the cyanate ester polymerization, the effective activation energy usually demonstrates a rather abrupt increase [30,31,32], which is obviously not the case in the present reaction (Figure 4A). Note that the absence of a transition to the diffusion-controlled regime is not unusual for cross-linking polymerization under nonisothermal conditions. This is because a decrease in molecular mobility due to network formation is compensated by its increase due to the continuously rising temperature. The transition is more common for isothermal conditions, under which a decrease in molecular mobility frequently results in incomplete polymerization, whereas under nonisothermal conditions polymerization tends to proceed to completion.

As a matter of fact, the observed *E_α_* dependence is rather common for parallel competing reactions [33]. Indeed, the polymerization of cyanate esters is commonly treated by the Kamal model (6) that combines parallel *n*th-order and auto-catalytic reactions [20,34,35,36,37,38,39,40,41]. 

Using the following form of the Kamal model:

(8) $$\frac{d\alpha }{dt}=A_{1}\exp\left(-E_{1}/RT\right){\left(1-\alpha \right)}^{n}+A_{2}\exp\left(-E_{2}/RT\right)\alpha^{m}{\left(1-\alpha \right)}^{n}\;\;\;$$

one can derive the theoretical dependence for the isoconversional activation energy expressed by Equation (9) [42]:

(9) $$E_{\alpha }=\frac{(A_{1}/A_{2})\exp(-E_{1}/RT)E_{1}+\alpha^{m}\exp(-E_{2}/RT)E_{2}}{\left(A_{1}/A_{2})\exp(-E_{1}/RT)+\alpha^{m}\exp(-E_{2}/RT\right)}\;$$

Fitting this theoretical dependence to to the experimentally detemined dependence of the effective activation energy (Figure 4A) permits determining the kinetic parameters of the individual steps of the solution polymerization, namely activation energies *E*_1_ and *E*_2_, the ratio of preexponential factors *A*_1_*/A*_2_, as well as autocatalytic reaction order *m*. We have assumed that the *nth*-order reaction governs the kinetics of the process at low conversions, where the concentration of the product is low, because *α^m^* is close to 0 and *E_α→0_ ≈ E*_1_. Thus, we have set the value of *E_1_* to 70 kJ mol^−1^. The values of other parameters *E*_2_, *A*_1_/*A*_2_, and *m* have been optimized in this fit (Table 1, first raw data). The next step of our kinetic analysis is the evaluation of the individual pre-exponential factors values *A_1_* and *A*_2_, as well as the reaction order *n*. To do that we fitted Equation (8) to the experimentally measured polymerization rates for all heating programs. The values of *E*_1_, *E*_2_, and *m* were not optimized in this fit and were kept equal to those found from fitting Equation (9). It should be noted that fitting Equation (8) while forcing the *A*_1_/*A*_2_ ratio obtained from Equation (9) results in poorer fits. Therefore, values *A*_1_ and *A*_2_ have been allowed to vary independently. The averaged values of the fitted parameters are collected in Table 1 (second row data). The results of the fitting of Equation (8) for each heating rate are presented in a Table S1 (See Supplementary Materials).

**Figure 5:**
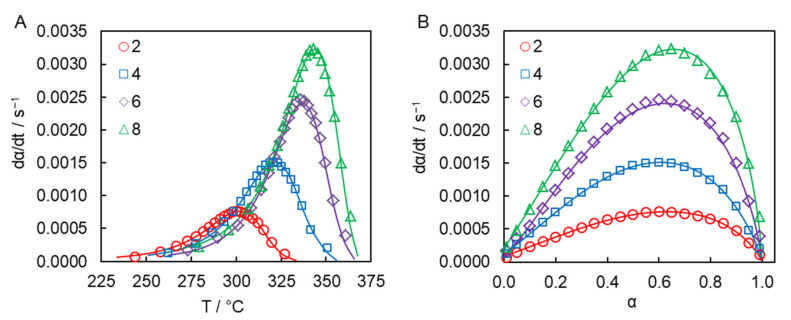
dα/dt vs. T (**A**) and dα/dt vs. α (**B**) experimental data (markers) and best fits to the Kamal model (solid lines) for the solution polymerization of dicyanate ester (numbers denote heating rates in °C min^−1^).

The ability of the obtained parameters of the Kamal model to reproduce experimental dependence of the polymerization rate on temperature and conversion is illustrated in Figure 5. 

**Figure 6:**
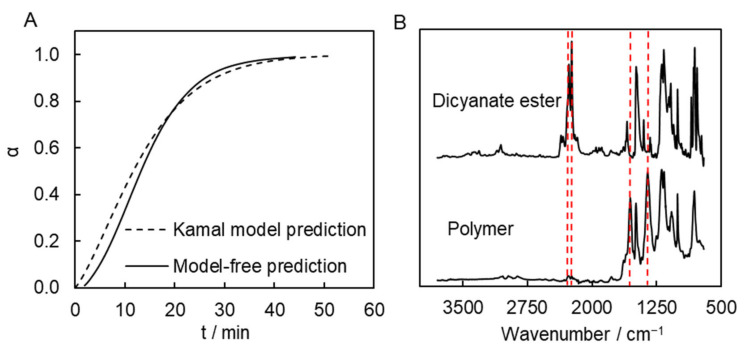
Predicted conversion-time curves for polymerization at 300 °C (**A**) and FTIR spectra of dicyanate ester and its polymerization product (**B**). Dashed lines represent absorption peaks at 2269, 2237, 1550, and 1350 cm^−1^.

The results of the above kinetic analyses were then used to predict the time to completion under isothermal conditions. This was done in a model-based way, i.e., by integrating the Kamal model (Equation (8)), as well as in a model-free way, i.e., by substituting the *E_α_*-dependence (Figure 4A) into Equation (6) [23]. In both cases, we looked for the temperature at which the process would be completed in roughly 1 h. This time is markedly shorter than the 20–40 h reported by other works on the preparation of microporous polytriazines [10,11,12,13,14]. Obviously, the faster procedure has the advantage of being more economical. On the other hand, one should avoid the synthetic procedure being too fast. This is because cyanate ester polymerization is highly exothermic [19], so that polymerization occurring too quickly may result in significant overheating of the sample, which can cause excessively rapid evaporation of the solvent and production of inferior quality material. The calculations suggest that the process would reach completion in roughly 1 h at a temperature of 300 °C. Namely, the Kamal model-based calculation predicts that α = 0.99 is achieved in ~51 min at this temperature. The model-free isoconversional calculation predicts that similar a conversion is reached in ~45 min at the same temperature (Figure 6A). That is, both approaches predict very similar times necessary for complete conversion.

These predictions were tested by performing polymerization at 300 °C in a muffle furnace. Polymerization was carried out in a sealed glass ampule flushed with argon. We also estimated the time it takes for the sample to go from 200 °C (initial sample temperature) to 300 °C (polymerization temperature). According to our estimation, this time is approximately 3–4 min. This time needs to be added to the predicted time. Thus, we polymerized the monomer solution at 300 °C for 55 min. A FTIR spectrum of the resulting synthesized polymer shows the presence of the absorption bands of triazine fragments (1550 and 1350 cm^−1^). Yet, the absorption bands corresponding to the unreacted cyanate ester groups (2269 and 2237 cm^−1^) are undetectable, which confirms that the polymerization process is complete (Figure 6B).

**Figure 7:**
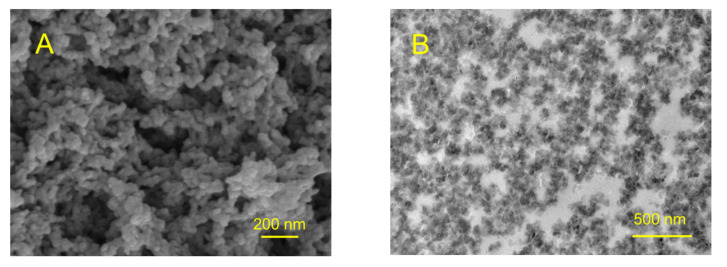
SEM image of the surface morphology of synthesized polymer (**A**) and TEM image of ultrathin cut of the synthesized polymer (**B**).

We also tested whether the synthesized material possesses a microporous structure. The surface morphology of the synthesized polymer sample was studied by SEM (Figure 7A), which shows that obtained material consists of polymeric grains with a size of ~50 nm. One can also see meso- and macro-pores of irregular shape formed within the material. The presence of meso- and macro-pores formed by polymeric grains is also clearly observed in the TEM image of an ultrathin cut of the polymer (Figure 7B). 

**Figure 8:**
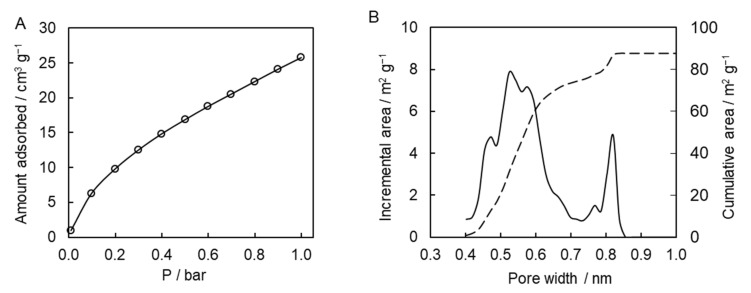
Adsorption isotherm for carbon dioxide at 0 °C (**A**); incremental and cumulative pore-size distribution curves (**B**).

To gain further insights into the porous system of the synthesized polymer, we used carbon dioxide adsorption porosimetry. This type of measurements is widely used for the characterization of microporous carbon-based solids, e.g., activated carbons and carbon molecular sieves [43]. The results of the adsorption measurements are presented in Figure 8A. The affinity of the presently studied polymer to carbon dioxide is comparable to the one for other porous polytriazines made of bi-, tri-, and tetra-functional monomers [10,11,13,14]. Calculations of pore-size distribution based on the adsorption data show that the synthesized polymeric material possesses multimodal distribution of micropores with two distinct major maxima at ~0.5 and 0.8 nm (Figure 8B). The micropore surface area is 88 m^2^ g^−1^, and the micropore volume is 0.03 cm^3^ g^−1^. Apparently, micropores were formed successfully in the resulting polymeric material. While beyond the objective of the present study, it is of interest for future work to explore the broad effect of the temperature conditions (isothermal and nonisothermal) and respective reaction kinetics on the porous structure of the final material. 

## 5. Conclusions

This study for the first time applies kinetic analysis to the problem of the synthesis of microporous materials via the polymerization of dicyanate ester in diphenyl sulfone. It shows that polymerization proceeds in a reaction-controlled regime and that the process kinetics can be parameterized by the Kamal reaction model. The process kinetics are also parameterized in a model-free way by using an advanced isoconversional method. Both approaches are capable of employing nonisothermal DSC measurements for predicting the isothermal kinetics at a given temperature. For the reaction under study, both approaches result in similar times for the completion of the polymerization. An independent isothermal test validates the correctness of the predictions. The porous structure of the obtained polymeric material is confirmed and characterized by electron microscopy and adsorption measurements. Overall, the study highlighted the value of nonisothermal kinetic methods in selecting the proper temperature conditions for the isothermal synthesis of microporous polymeric materials.

## Figures and Tables

**Table 1:** Estimated kinetic parameters of the Kamal model for polymerization of dicyanate ester.

Equation	*E*_1_/kJ mol^−1^	*E*_2_/kJ mol^−1^	*A*_1_/s^−1^	*A*_2_/s^−1^	*A*_1_ */A*_2_	*m*	*n*	*R* ^2^
Equation (9)	70 *	145 ± 5	-	-	(3 ± 2) × 10^−7^	0.53 ± 0.04	-	0.99
Equation (8)	70 *	145 *	(9 ± 2) × 10^2^	(2.3 ± 0.7) × 10^10^	-	0.53 *	1.0 ± 0.1	0.99

* denotes values fixed during the fittings.

## Data Availability

The data presented in this study are available on request from the corresponding authors.

## Supplementary Materials

The following supporting information can be downloaded at: https://www.mdpi.com/article/10.3390/molecules27113605/s1, Table S1. Results of the fitting of the Kamal model to experimental data.
