# Supplementary material for: The Kinetics of Formation of Microporous Polytriazine in Diphenyl Sulfone
Source: Molecules. 2022 Jun 3;27(11):3605. doi: 10.3390/molecules27113605 (PMC9181875; doi:10.3390/molecules27113605)
Supplement: Supplementary file 1 [file molecules-27-03605-s001.zip › molecules-1733857-supplementary.pdf]

Supporting Information

The kinetics of formation of microporous polytriazine in diphenyl sulfone

Andrey Galukhin[1,*], Ilya Nikolaev [1], Roman Nosov [1], and Sergey Vyazovkin [2,*]

1 Alexander Butlerov Institute of Chemistry, Kazan Federal University, Kremlevskaya Str. 18, 420008 Kazan, Russia; romanosow@mail.ru (R.N.); ilkamoe1995@yandex.ru (I.N.)
2 Department of Chemistry, University of Alabama at Birmingham, 901 S. 14th Street, Birmingham, AL 35294, USA
* Correspondence: and_galuhin@mail.ru (A.G.); vyazovkin@uab.edu (S.V.)

Table S1. Results of the fitting of the Kamal model to experimental data.

Heating rate / °C min^{-1}	E_1/kJ mol^{-1}	E_2/kJ mol^{-1}	A_1/s^{-1}	A_2/s^{-1}	m	n	R^2
2	70*	145*	(1.03±0.02) ×10^3	(3.15±0.05) ×10^{10}	0.53*	1.00±0.01	0.99
4			(0.86±0.02) ×10^3	(2.56±0.02) ×10^{10}		1.09±0.01	0.99
6			(0.60±0.02) ×10^3	(1.88±0.02) ×10^{10}		0.95±0.01	0.99
8			(1.00±0.03) ×10^3	(1.60±0.02) ×10^{10}		0.84±0.01	0.99

*denotes values fixed during the fittings.
